# Quantitative Proteomics Reveals Metabolic Reprogramming in Host Cells Induced by Trophozoites and Intermediate Subunit of Gal/GalNAc Lectins from Entamoeba histolytica

**DOI:** 10.1128/msystems.01353-21

**Published:** 2022-03-28

**Authors:** Yanqing Zhao, Xia Li, Ruixue Zhou, Lei Zhang, Lijun Chen, Hiroshi Tachibana, Meng Feng, Xunjia Cheng

**Affiliations:** a Department of Medical Microbiology and Parasitology, School of Basic Medical Sciences, Fudan University, Shanghai, China; b Affiliated Hospital of Guizhou Medical University, Guiyang, China; c Institutes of Biomedical Sciences, Fudan University, Shanghai, China; d Department of Infectious Diseases, Tokai University School of Medicine, Isehara, Kanagawa, Japan; Princeton University; University of Guelph

**Keywords:** *Entamoeba histolytica*, Gal/GalNAc lectins, quantitative proteomics, metabolic reprogramming, pyruvate kinase

## Abstract

Entamoeba histolytica is an intestinal protozoan parasite with remarkable ability to kill and phagocytose host cells, causing amoebic colitis and extraintestinal abscesses. The intermediate subunit (Igl) of galactose (Gal)- and *N*-acetyl-d-galactosamine (GalNAc)-specific lectins is considered an important surface antigen involved in the pathogenesis of E. histolytica. Here, we applied mass spectrometry-based quantitative proteomics technology to analyze the protein expression profile changes occurring in host Caco2 cells incubated with E. histolytica trophozoites or stimulated by purified native Igl protein. The expression levels of 1,490 and 489 proteins were significantly altered in the E. histolytica-treated and Igl-treated groups, respectively, among 6,875 proteins totally identified. Intriguingly, central carbon metabolism of host cells was suppressed in both E. histolytica-treated and Igl-treated groups, with evidence of decreased expression levels of several key enzymes, including pyruvate kinase muscle type 2, presenting a Warburg-like effect in host cells. Besides, Igl had potential physical interactions with central carbon metabolism enzymes and the proteolytic degradation family members proteasome subunit alpha and beta, which may be responsible for the degradation of key enzymes in carbon metabolism. These results provided a novel perspective on the pathogenic mechanism of E. histolytica and compelling evidence supporting the important role of Igl in the virulence of E. histolytica.

**IMPORTANCE** Metabolic reprogramming is considered a hallmark of some infectious diseases. However, in amoebiasis, a neglected tropical disease caused by protozoan parasite E. histolytica, metabolic changes in host cells have yet to be proven. In this study, advanced data-independent acquisition mass spectrometry-based quantitative proteomics was applied to investigate the overall host cellular metabolic changes as high-throughput proteomics could measure molecular changes in a cell or tissue with high efficiency. Enrichment analysis of differentially expressed proteins showed biological processes and cellular pathways related to amoeba infection and Igl cytotoxicity. Specifically, central carbon metabolism of host cells was dramatically suppressed in both E. histolytica-treated and Igl-treated groups, indicating the occurrence of a Warburg-like effect induced by trophozoites or Igl from E. histolytica. Distinct differences in ubiquitin-mediated proteolysis, rapamycin (mTOR) signaling pathway, autophagy, endocytosis, and tight junctions provided novel perspectives on the pathogenic mechanism of E. histolytica.

## INTRODUCTION

Entamoeba histolytica is an enteric protozoan parasite with a remarkable ability to kill cells and cause amoebiasis. The hallmark of amoebiasis is profound tissue destruction, which results in massive intestinal ulceration and even fatal extraintestinal abscesses. It has been estimated that 50 million people suffer from invasive amoebiasis worldwide, causing approximately 100,000 deaths annually (1–4). The adherence of E. histolytica trophozoites to colonic mucins and epithelial cells is the initial event of colonization and invasion and is an indispensable prerequisite for amoebic cytotoxicity (5). Adherence is mainly mediated by a group of galactose (Gal)- and *N*-acetyl-d-galactosamine (GalNAc)-specific lectins present on the plasma membrane of E. histolytica trophozoites (6, 7).

At the early stage of amoebic infection, trophozoites can kill host cells using Gal/GalNAc lectins and other secreted toxic effectors, such as amoebapore (8) and cysteine proteases (9). The Gal/GalNAc lectins from E. histolytica are comprised of a 260-kDa heterodimer and a 150-kDa intermediate subunit (Igl) linked by noncovalent bonds. Igl is considered an important surface antigen that can elicit immunological responses in hosts (5, 10, 11). Studies have shown that Igl seems to be closely associated with the pathogenicity of E. histolytica: antibodies against Igl could block amoebic adherence to host cells *in vitro* and prevent amoebic liver abscess formation *in vivo* (12, 13). Igl can also work with elongation factor-1 alpha to facilitate amoebic phagocytosis (14). Besides, amoebic trogocytosis can lead to irreversible intracellular calcium elevation in human cells and ultimately cell death (15, 16).

Previous studies on the pathogenicity of E. histolytica have primarily focused on the biological behavior of the trophozoites. However, the specific mechanisms and processes underlying the metabolic changes in host cells upon E. histolytica infection remain poorly understood. Meanwhile, in the process of amoebic infection, how Igl, an important subunit of the Gal/GalNAc lectins, contributes to metabolic reprogramming in host cells remains elusive. Metabolic reprogramming is an important sign of host-pathogen interactions (17–19). Rewiring of important metabolic pathways and manipulation of cell signaling pathways by parasites have been demonstrated to be associated with cell survival and proliferation (20–22). Hence, investigation of the metabolic changes occurring in host cells could help us better understand the pathogenicity of E. histolytica. Taking these needs into account, the present study assessed the molecular changes in Caco2 cells, a human epithelial cell line that has been widely used as a model of the intestinal epithelial barrier and is thus suitable as a host cell for E. histolytica (23–25), after E. histolytica trophozoites or Igl stimulation using a mass spectrometry-based, data-independent acquisition (DIA) quantitative proteomics approach.

## RESULTS

### Quantitative proteomics analysis of protein expression profiles in host cells treated with E. histolytica or Igl.

**FIG 1:**
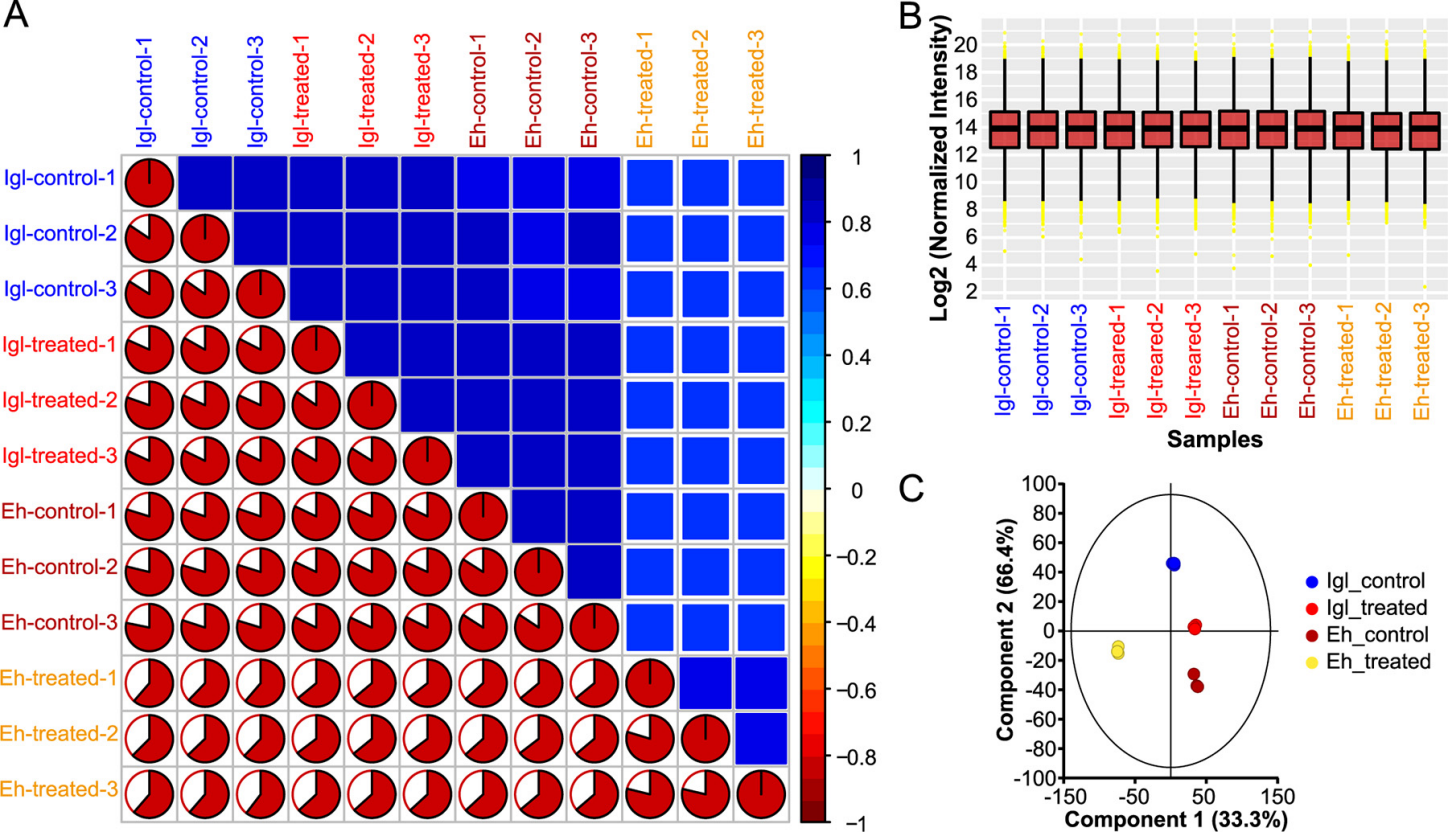
Quality control of the mass spectrometry platform and overview of protein expression landscape. (A) Correlations between peak areas of any two samples from four groups. The bottom-left half of the panel represents the pairwise Pearson correlation coefficients of the samples, and the top-right half of the panel depicts the pairwise scatterplots from the same comparison. (B and C) Average for differentially expressed genes (B) and principal-component analysis (C) in the four groups analyzed in quantitative proteomics. Igl control group, Igl-treated group, E. histolytica control group, and E. histolytica-treated group are marked in blue, red, brown, and yellow, respectively.

Caco2 cells were cultured under four different conditions: incubation with trophozoites of E. histolytica with amoeba medium, amoeba medium only, purified native Igl protein solubilized in cell medium, or cell medium only. We extracted whole-protein samples from the prepared Caco2 cells and applied a mass spectrometry-based, DIA quantitative proteomics approach to elucidate the molecular changes occurring in host cells treated with E. histolytica or Igl protein. The reproducibility of the results was evaluated by calculating the pairwise Pearson correlation coefficients of peak areas between any two samples from the four groups. The adopted liquid chromatography-tandem mass spectrometry (LC-MS/MS) analysis showed excellent repeatability and reliability in the identification and quantification of protein expression profiles in different samples (Fig. 1A). The intensity normalization procedure was performed as shown in Fig. 1B. Principal-component analysis (PCA) showed clear stratification among the different groups (Fig. 1C).

A total of 6,875 human proteins were identified from all groups. The number of proteins identified was valid value-filtered for peptide spectrum matches (false-discovery rate [FDR] = 1%) and for protein group (FDR = 0.9%). Nearly three times more genes in E. histolytica-treated cells (1,490 genes, Log2FC [fold change] < −0.5 or Log2FC > 0.5, *P* < 0.05 in Student’s *t* test with FDR correction) were upregulated or downregulated than those in Igl-treated cells (489 genes, Log2FC < −0.5 or Log2FC > 0.5, *P* < 0.05 in Student’s *t* test with FDR correction). This result indicated that E. histolytica infection can cause profound metabolic changes as well as cell signaling changes in host cells and that Igl could play a role in the pathogenesis of E. histolytica.

**FIG 2:**
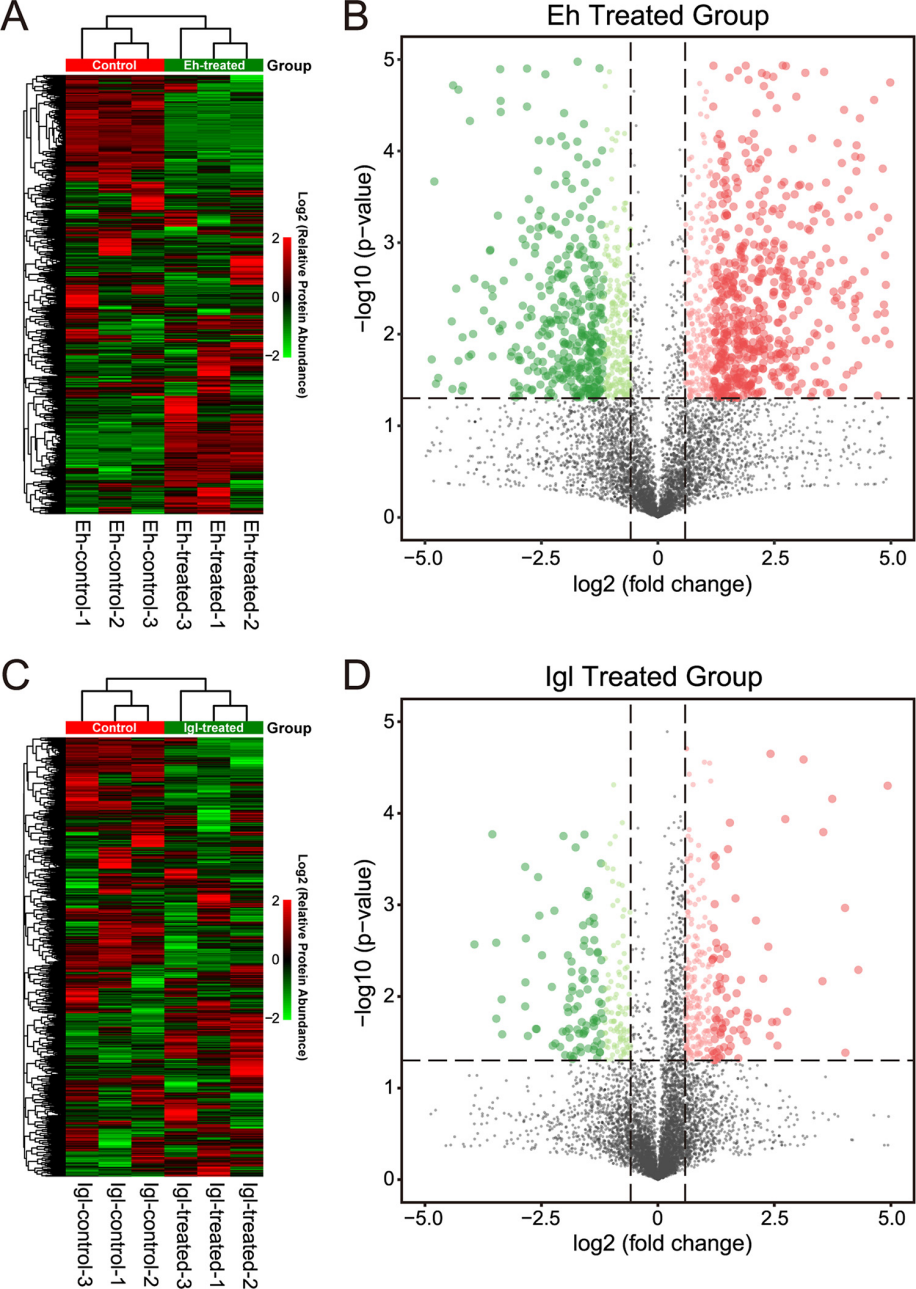
Quantitative proteomics analysis of Caco2 cells after infection with E. histolytica or stimulation by Igl. Heat map (A) and volcano plots (B) of differentially expressed genes in Caco2 cells stimulated with E. histolytica. Heat map (C) and volcano plots (D) of differentially expressed genes in Caco2 cells stimulated with Igl. Upregulated genes are shown in red, and downregulated genes are shown in green.

In the E. histolytica trophozoite-treated group, the expression levels of 1,490 proteins were significantly changed compared to those in the control group, among which 607 (8.8% of total) were upregulated and 883 (12.8% of total) were downregulated (Log2FC < −0.5 or Log2FC > 0.5, *P* < 0.05 in Student’s *t* test) (Fig. 2A and B). In the Igl-treated group, the expression levels of 489 proteins were significantly changed compared to the control group, among which 289 (4.2% of total) were upregulated and 200 (2.9% of total) were downregulated (Log2FC < −0.5 or Log2FC > 0.5, *P* < 0.05 in Student’s *t* test) (Fig. 2C and D). The differential protein expression profiles indicated that obvious molecular changes occurred in host cells after incubation with E. histolytica trophozoites or Igl protein.

### Heterogeneous regulation of protein expression in host cells infected with E. histolytica or Igl.

Gene Ontology (GO) enrichment analysis was used to determine the biological process (BP), cell component (CC), and molecular function (MF) of the differentially expressed proteins detected by quantitative proteomics analysis, and Kyoto Encyclopedia of Genes and Genomes (KEGG) enrichment analysis was used to evaluate the general molecular landscape associated with E. histolytica trophozoites and Igl protein.

**FIG 3:**
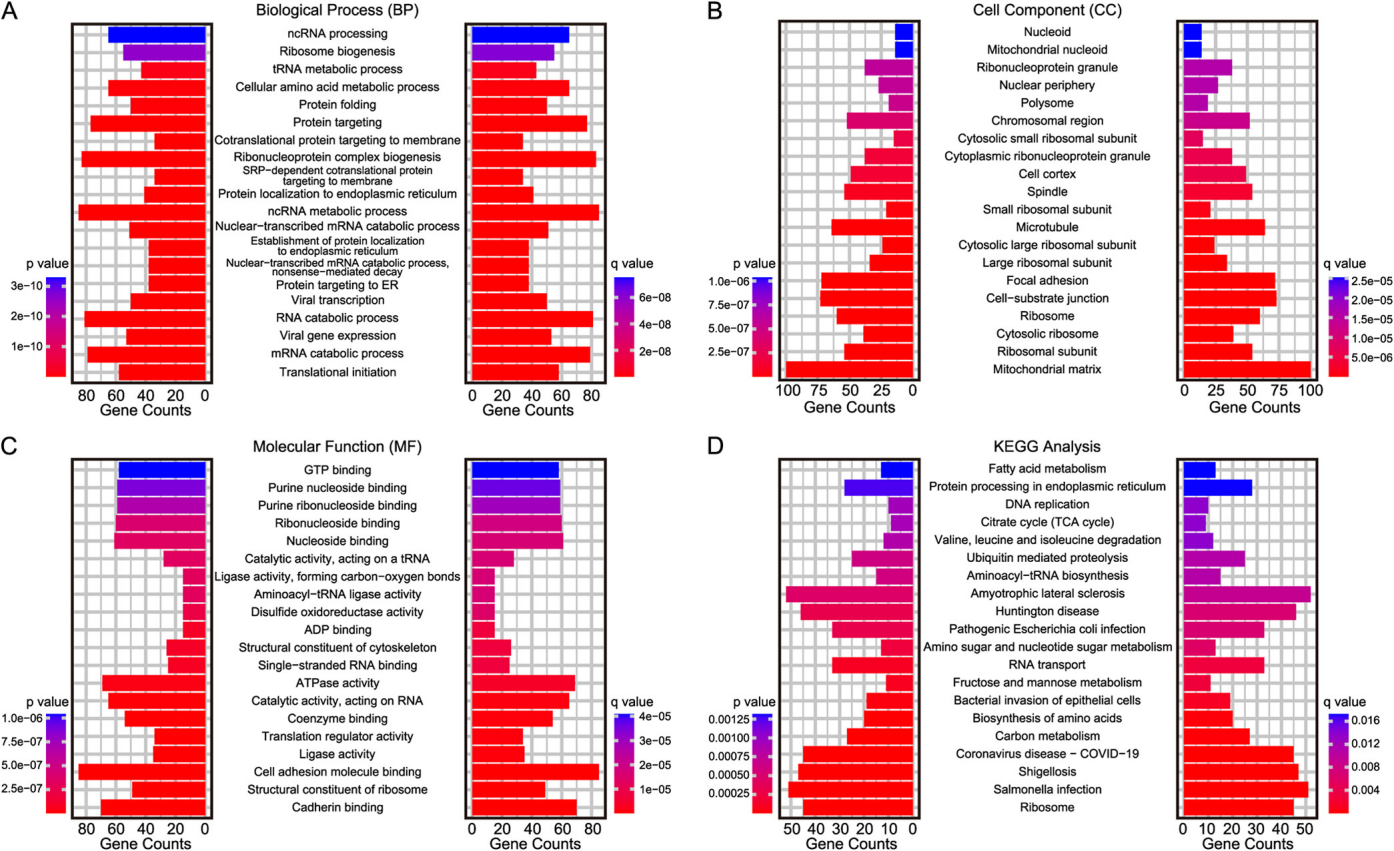
Enrichment analysis of altered expressed proteins in Caco2 cells infected with E. histolytica. Gene Ontology (GO) and Kyoto Encyclopedia of Genes and Genomes (KEGG) enrichment analysis of differentially expressed proteins in Caco2 cells infected with E. histolytica, including the enriched clusters of biological process (A), cell components (B), molecular function (C), and KEGG pathways (D). TCA, tricarboxylic acid.

In the E. histolytica-treated group, 65 pathways were significantly altered (*P* < 0.05, Student’s *t* test). In particular, E. histolytica trophozoites had a strong impact on carbon metabolism, citrate cycle, amino sugar and nucleotide sugar metabolism, and protein translation and targeting. The differentially expressed proteins gathered mainly in mitochondria and ribosomes, indicating that E. histolytica trophozoites could cause metabolic reprogramming in host cells. Interestingly, pathways related to infectious diseases, such as Salmonella infection, shigellosis, and coronavirus disease, were also significantly altered in the E. histolytica-treated group (Fig. 3).

**FIG 4:**
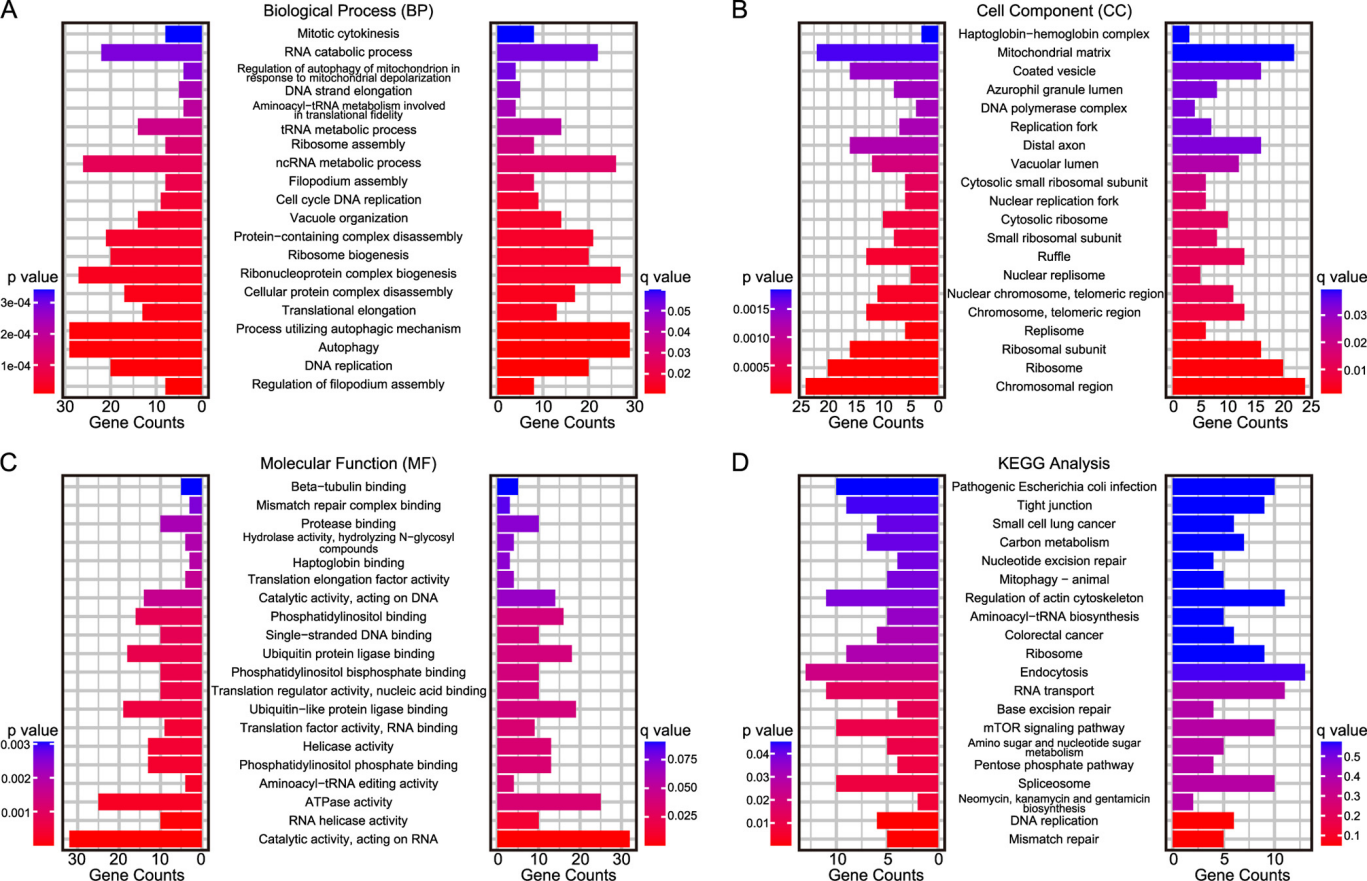
Enrichment analysis of altered expressed proteins in Caco2 cells stimulated with Igl. GO and KEGG enrichment analysis of differentially expressed proteins in Caco2 cells stimulated with Igl, including enriched clusters of biological process (A), cell components (B), molecular function (C), and KEGG pathways (D).

In the Igl-treated group, 20 pathways were significantly altered (*P* < 0.05, Student’s *t* test). Igl mainly affected carbon metabolism, the mammalian target of rapamycin (mTOR) signaling pathway, and autophagy of host cells. The differentially expressed proteins were mainly located in the chromosomal region and ribosomes, indicating that Igl may act as a signal molecule affecting the gene expression profile of host cells. Meanwhile, Igl may be involved in metabolic reprogramming caused by E. histolytica trophozoites (Fig. 4).

### Metabolic reprogramming of host cells induced by E. histolytica and Igl.

Observing the similarities between the E. histolytica-treated group and Igl-treated group, the central carbon metabolism was significantly affected in both groups. Cellular metabolism is important to cells due to its role of energy generation through glycolysis and oxidative phosphorylation. The processes of cellular metabolism are catalyzed by multiple enzymes, and the rate of cellular respiration is regulated by the content and activity of several key enzymes, including pyruvate kinase (PKM), ATP-dependent 6-phosphofructokinase (PFKM), 6-phosphogluconolactonase (PGLS), succinyl coenzyme A (succinyl-CoA) synthetase (SUCLG1), phosphoglycerate kinase 1 (PGK1), and dihydrolipoyllysine-residue acetyltransferase component of pyruvate dehydrogenase complex (DLAT). Changes in cellular metabolic status, namely, metabolic reprogramming, are often considered to be closely associated with infections and other diseases.

**FIG 5:**
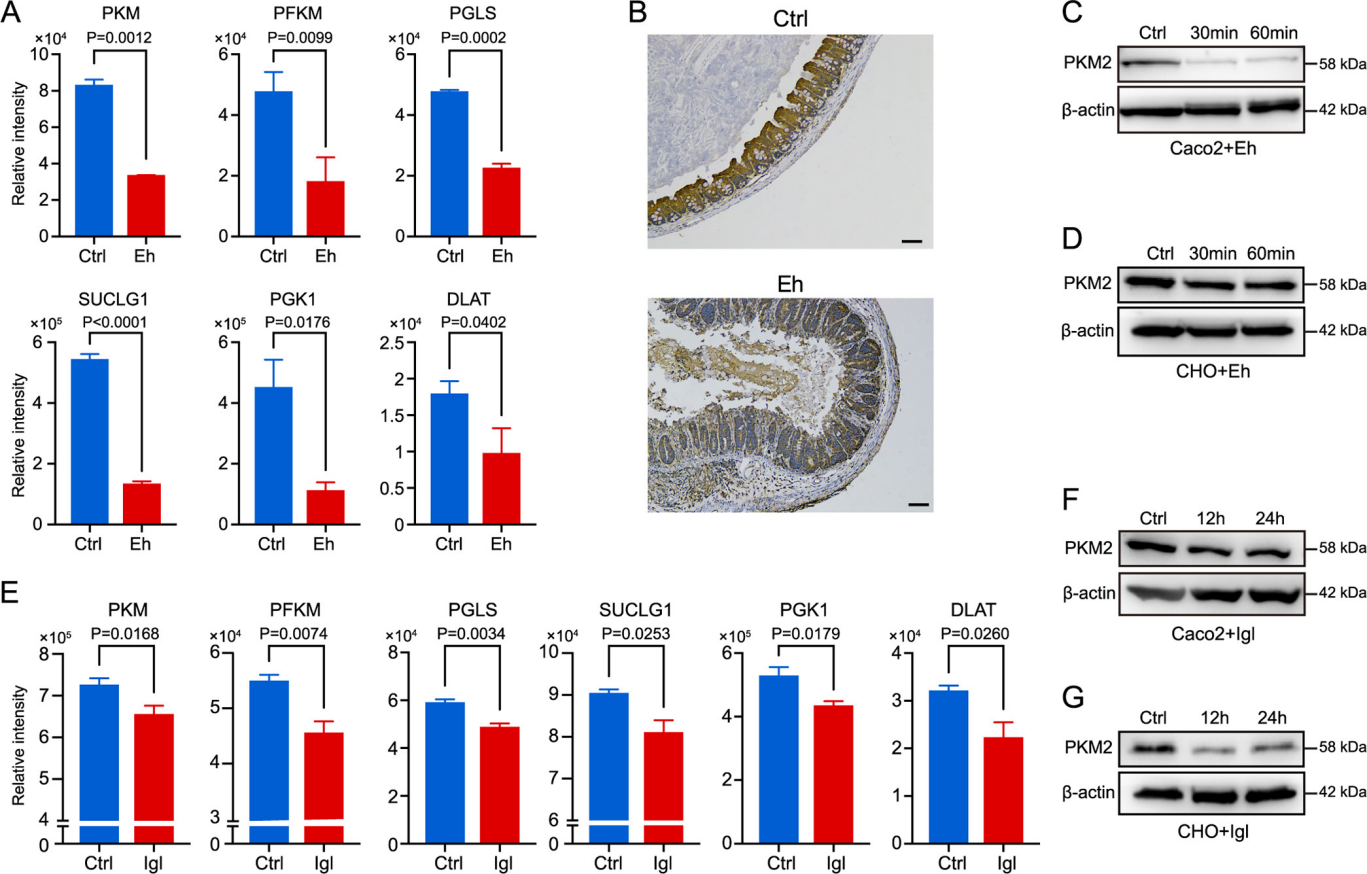
Metabolic reprogramming of host cells induced by E. histolytica trophozoites and purified Igl protein. (A) Bar charts display the expression changes of proteins associated with central carbon metabolism induced by E. histolytica trophozoites (Student’s *t* test), including pyruvate kinase (PKM), ATP-dependent 6-phosphofructokinase (PFKM), 6-phosphogluconolactonase (PGLS), succinyl-CoA synthetase (SUCLG), phosphoglycerate kinase 1 (PGK1), and dihydrolipoyllysine-residue acetyltransferase component of pyruvate dehydrogenase complex (DLAT). (B) Representative immunohistochemical images showing that PKM2 was decreased in intestinal tissue from the mouse model of amoebic colitis. Scale bar, 100 μm. Western blotting showed that PKM2 was decreased in both Caco2 cells (C) and CHO cells (D) after 0- (control), 30-, or 60-min E. histolytica stimulation. (E) Bar charts display the expression changes of proteins associated with central carbon metabolism induced by purified Igl protein (Student’s *t* test), including PKM, PFKM, PGLS, SUCLG1, PGK1, and DLAT. (F and G) Western blotting showed that PKM2 was decreased in both Caco2 cells (F) and CHO cells (G) after 12- or 24-h Igl stimulation. Control: Caco2 or CHO cells without Igl stimulation.

In the E. histolytica-treated group, protein expression levels of PKM, PFKM, PGLS, SUCLG1, PGK1, and DLAT were dramatically decreased (Fig. 5A), indicating the suppression of the central carbon metabolism in host cells incubated with E. histolytica trophozoites. To confirm that metabolic reprogramming occurred in host cells, immunohistochemical staining of intestinal tissue from a mouse model of amoebic colitis was performed. The results showed that pyruvate kinase muscle type 2 (PKM2) was significantly decreased in the E. histolytica-infected colons compared with that in the control group (Fig. 5B and see also Fig. S1 in the supplemental material). In addition, Western blotting showed that the protein expression levels of PKM2 were decreased in both Caco2 cells (Fig. 5C) and Chinese hamster ovary (CHO) cells (Fig. 5D) after incubation with E. histolytica trophozoites.

10.1128/msystems.01353-21.1 FIG S1 Histopathology of intestinal tissue from the mouse model of amoebic colitis. The intestinal tissue was stained by hematoxylin and eosin (HE) or periodic acid-Schiff (PAS) stain. Microscope magnification, ×100 and ×400; scale bar, 200 μm and 50 μm. Arrows indicate E. histolytica trophozoites. Download FIG S1, TIF file, 2.8 MB. Copyright © 2022 Zhao et al. 2022 Zhao et al. https://creativecommons.org/licenses/by/4.0/ This content is distributed under the terms of the Creative Commons Attribution 4.0 International license.

Consistent with those in the E. histolytica-treated group, the protein expression levels of PKM, PFKM, PGLS, SUCLG1, PGK1, and DLAT were also significantly decreased in the Igl-treated group (Fig. 5E), indicating the metabolic reprogramming induced by purified Igl protein. Western blotting was performed to confirm the decrease of PKM2 in both Caco2 cells (Fig. 5F) and CHO cells (Fig. 5G) after Igl stimulation.

**FIG 6:**
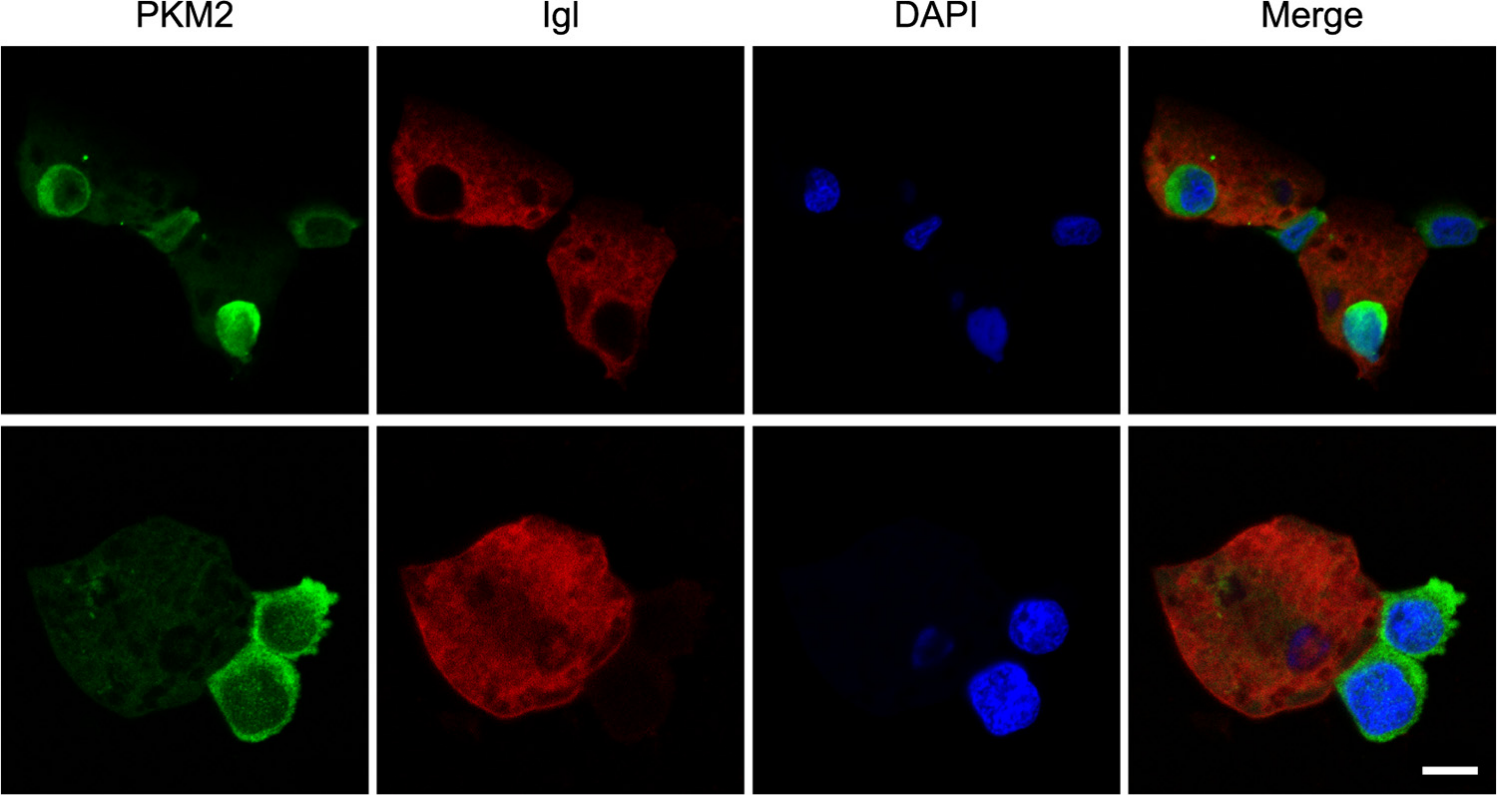
Representative confocal images of host cells incubated with E. histolytica trophozoites for 30 min. Green, PKM2; red, Igl; blue, DAPI. Scale bar, 10 μm.

Furthermore, confocal microscopy was conducted to observe the expression level and cellular location of PKM2. Results showed that most host cells incubated with trophozoites had relatively small amounts of PKM2, but individual cells showed the opposite trend, with PKM2 aggregated at the contact sites of host cells and trophozoites (Fig. 6), suggesting that PKM2 participated in this intercellular interaction.

### Interaction between Igl and host cell proteins.

**FIG 7:**
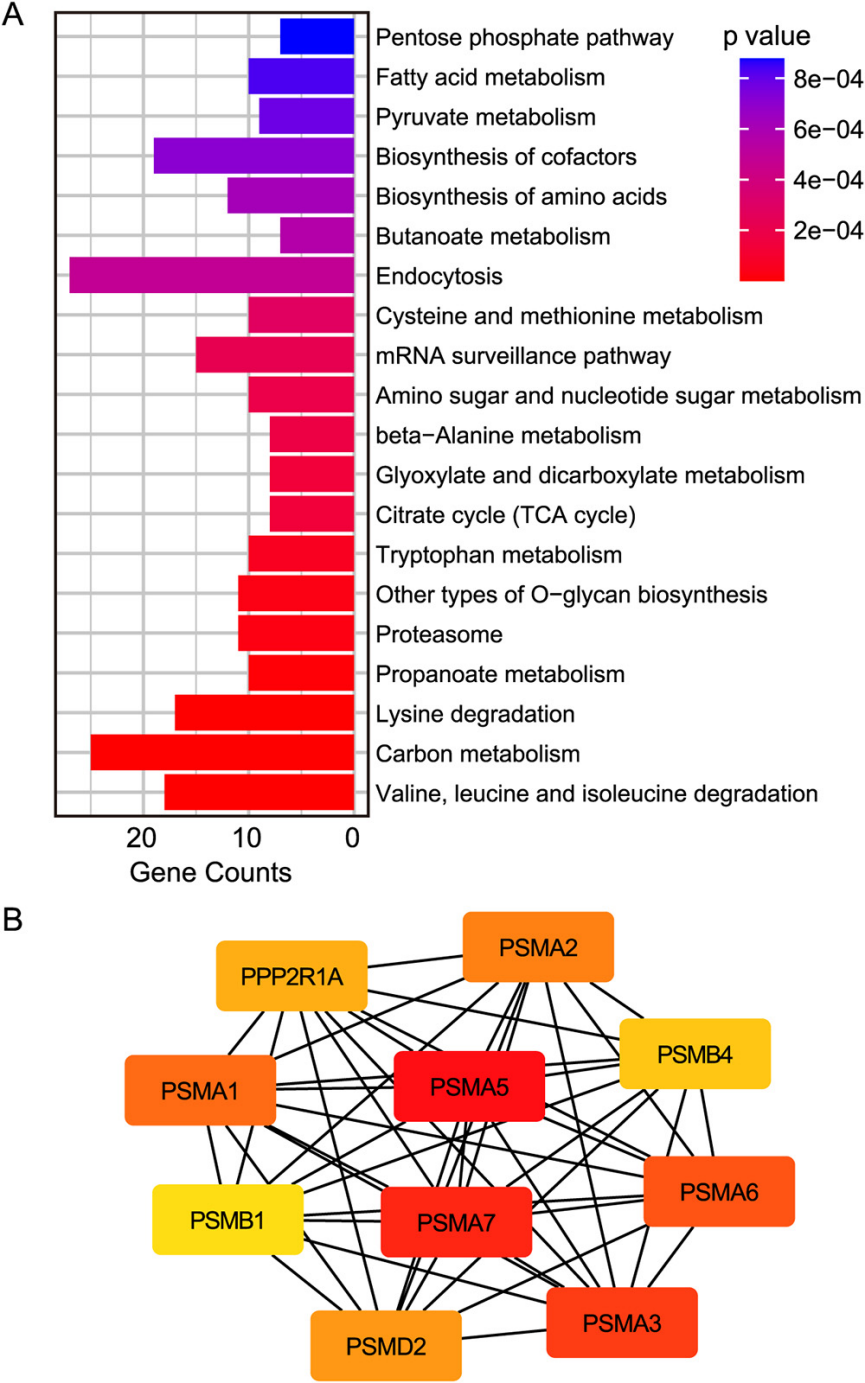
Functional analysis of proteins obtained by Igl pulldown assay. (A) KEGG enrichment analysis of proteins obtained by Igl pulldown assay. (B) The top 10 hub genes identified in the protein-protein interaction network.

A protein pulldown assay was performed to evaluate the interaction between Igl and proteins from the host cells. Protein mass spectrum analysis showed that 801 proteins involved in pathways related to carbon metabolism, the citrate cycle, pyruvate metabolism, and endocytosis might interact with the purified Igl protein (Fig. 7A). These host proteins may also interact with each other and form a functional network, which could be regulated by Igl. To further explore the impacts of Igl stimulation inside host cells, interaction networks of Igl-associated host cell proteins were constructed using STRING. Moreover, the interactors of the top 10 hub genes were built using Cytoscape software and its plugin cytoHubba, and these genes were the most important nodes in the protein-protein interaction network. According to the cytoHubba Maximal Clique Centrality ranking, eight of the top 10 hub genes (*Proteasome subunit alpha (PMSA) 5*, *PSMA7*, *PSMA3*, *PSMA6*, *PSMA1*, *PSMA2*, *Proteasome subunit beta (PMSB) 4*, and *PSMB1*) were subtypes of proteasomes, which are responsible for the proteolytic degradation of most intracellular proteins (Fig. 7B). This result suggested the potential physical interactions among Igl, proteasome family members, including PSMA and PSMB subunits, and proteins related to carbon metabolism.

### Additional important signaling pathways affected by E. histolytica and Igl.

We next examined the expression changes in particular groups of genes related to the pathogenesis of E. histolytica trophozoites and Igl from proteomics data. The data are presented as Log2FC of expression and *P* value. Functional annotation clustering was performed to categorize the genes into classes like bacterial invasion of epithelial cells and ubiquitin-mediated proteolysis in E. histolytica-treated cells (Fig. S5A), mTOR signaling pathway, autophagy, endocytosis, pathogenic Escherichia coli infection, and tight junctions in Igl-treated cells (Fig. S5B). The results showed that Igl could activate mTOR, with evidence of increased expression levels of Rictor, Seh1l, and Lamtor2. mTOR is a key regulator of cellular metabolism in cells and of autophagy. Our results showed that autophagy was promoted in the Igl-treated group. Western blotting showed that the expression levels of autophagy marker LC3 increased in Igl-treated Caco2 cells, confirming the activation of autophagy by Igl stimulation (see Fig. S2). Finally, our data also indicated that Igl could disturb tight junctions between intestinal epithelial cells with decreased protein levels of MYH9, ARPC5L, and MICALL2 (Fig. S5B).

10.1128/msystems.01353-21.2 FIG S2 Western blotting of LC3 expression in Igl-stimulated Caco2 cells. Confirmation of increased expression levels of LC3 in Caco2 cells at 12 h and 24 h after Igl stimulation detected by Western blotting. Download FIG S2, TIF file, 2.2 MB. Copyright © 2022 Zhao et al. 2022 Zhao et al. https://creativecommons.org/licenses/by/4.0/ This content is distributed under the terms of the Creative Commons Attribution 4.0 International license.

10.1128/msystems.01353-21.5 FIG S5 Expression changes in particular groups of genes related to the pathogenesis of E. histolytica and Igl. Differentially expressed genes in the E. histolytica-treated group (A) and Igl-treated group (B). Download FIG S5, TIF file, 1.5 MB. Copyright © 2022 Zhao et al. 2022 Zhao et al. https://creativecommons.org/licenses/by/4.0/ This content is distributed under the terms of the Creative Commons Attribution 4.0 International license.

## DISCUSSION

The colon is the first tissue to be destroyed by E. histolytica trophozoites in the human body. The destruction of the colonic protective barrier and epithelial cells causes blood metastasis of trophozoites, which may result in potentially life-threatening diseases, such as amoebic liver abscess and brain abscess. The adhesion of amoebic trophozoites to colonic epithelial cells mainly depends on the Gal/GalNAc lectins (5, 11, 26). Once amoeba trophozoites adhere to host cells, a series of amoebic reactions are triggered, including the release of amoebapore, cysteine proteases, and peroxiredoxin, as well as trogocytosis, contributing to cell killing and tissue invasion (8, 9, 15, 27, 28). Our research lines focus on how host cells respond before apoptosis or phagocytosis induced by E. histolytica trophozoites. Specifically, what kind of toxicity or interference effects would Igl, an important subunit of Gal/GalNAc lectins, elicit during the early stage of infection?

Over the years, host-parasite interactions have been studied extensively from both the host and parasite aspects. Proteomics studies have provided new insights into host-pathogen cross talk (20, 29–31). Data-dependent acquisition (DDA) and DIA are both experimental paradigms in bottom-up proteomics. However, DIA can address some limitations of the conventional DDA strategy, for example, the bias in intensity-dependent precursor selection and limited dynamic range. These advantages, together with the recent developments in speed, sensitivity, and resolution in MS technology, position DIA as a great alternative to DDA (32, 33). In this study, we applied advanced DIA mass spectrometry-based quantitative proteomics technology to analyze protein expression changes in host Caco2 cells incubated with E. histolytica trophozoites or stimulated by Igl protein. In the E. histolytica-treated group, one of the most differentially regulated pathways was central carbon metabolism, indicating the occurrence of metabolic reprogramming induced by E. histolytica. Moreover, oxidative phosphorylation was suppressed in E. histolytica-infected cells despite the supply of abundant oxygen. This was similar to the well-studied Warburg effect occurring in cancer (34, 35). However, glycolysis was simultaneously suppressed in E. histolytica-treated host cells in this case. Studies have shown that central carbon metabolism in early prostate cancer is inhibited and that ATP production depends on lipids and other biomolecules (36). The Warburg effect, though ubiquitous in different cancers, does not occur in the initial stages of tumor development but becomes a prominent metabolic route when multiple mutations accumulate during tumor development (37). The Warburg-like effect was also observed in *Theileria*-transformed leukocytes with increased glucose uptake and fermentation of glucose to lactate (21, 38). These data showed that protozoan infection could impair the carbon metabolism of host cells, suggesting that early medical intervention might help host cells to reduce the burden of energy production.

Igl is a cysteine-rich protein located on the cell membrane of trophozoites. Under cysteine-deficient conditions, E. histolytica can strategically downregulate the expression of Igl, resulting in decreased amoebic phagocytosis and cytotoxicity (39). In addition, Igl-immunized hamsters showed increased expression levels of cytokines, such as interleukin-4 (IL-4) and IL-10, and the generated anti-Igl-specific antibodies could protect hamsters from the formation of amoebic liver abscess (13). These studies report that Igl is enormously involved in the pathogenesis of E. histolytica. In our present study, intriguingly, Igl-treated host cells showed decreased central carbon metabolism, similar to that in the E. histolytica-treated group. This finding suggested that purified Igl protein could induce metabolic reprogramming comparable to that induced by E. histolytica trophozoites, a novel mechanism of Igl in E. histolytica pathogenicity.

Protein pulldown assay is an important approach for studying protein-protein interactions. This assay indicated that the possible protein partners of Igl in host cells, including PSMA and PSMB (members of the proteolytic degradation family), might be involved in central carbon metabolism. Hence, we hypothesized that Igl could cause targeted degradation of proteins in the central carbon metabolism pathway through PSMA and PSMB. This could be one of the possible explanations for the observed degradation of several enzymes involved in carbon metabolism in proteomics studies. This hypothesis entailed a possible routine of cytolysis, during which Igl could degrade host cells or tissues without the participation of cysteine protease.

In addition to central carbon metabolism, other differentially expressed pathways in E. histolytica- or Igl-treated groups might indicate the pathogenesis of E. histolytica and functions of Igl. KEGG pathway enrichment analysis revealed that the pathogenesis of E. histolytica had some common molecular features with Salmonella infection, shigellosis, pathogenic Escherichia coli infection, and even coronavirus disease, suggesting that infectious intestinal diseases might share similar molecular pathology. In this study, Igl was shown to have effects on autophagy, the mTOR signaling pathway, and tight junctions. Autophagy is closely connected with the pathogenesis and inflammatory reactions of E. histolytica (30), and our results indicated that Igl could activate the mTOR signaling pathway and affect downstream processes, such as autophagy. In addition, tight junctions are intercellular junctions that build the epithelial barrier, and their dysfunction is closely associated with multiple local and systemic diseases (40, 41). Previous studies have shown that E. histolytica cysteine protease can damage the tight junctions of the intestinal epithelium, thereby promoting amoebic invasion (42, 43). Our results implied that Igl could also participate in the process of tight junction degradation.

In conclusion, our data showed that E. histolytica substantially suppressed central carbon metabolism in host epithelial cells with the participation of Igl. Igl is highly involved in E. histolytica-host interactions and promotes the virulence of E. histolytica by regulating the carbon metabolism of host cells. In future research, quantitative proteomics analysis of E. histolytica-infected tissues from animal models of amoebiasis could be performed to explore more specific mechanisms of metabolic reprogramming to comprehend the nature of amoebiasis.

## MATERIALS AND METHODS

### Cell culture.

Trophozoites of E. histolytica HM-1:IMSS and SAW755CR were grown in BI-S-33 medium containing 10% adult bovine serum (Sigma-Aldrich, St. Louis, MO, USA) at 36.5°C. CHO cells were grown in Ham’s F-12 medium (Corning, catalog no. 10-080-CV; Manassas, VA, USA) containing 10% fetal bovine serum (HyClone Laboratories, SH30396.03; Logan, UT, USA) at 37°C in a 5% CO_2_ incubator. Caco2 human epithelial cells were grown in Eagle minimum essential medium (MEM) (Corning, catalog no. 10-010-CV; Manassas, VA, USA) supplemented with 10% fetal bovine serum (HyClone Laboratories, SH30396.03; Logan, UT, USA), MEM nonessential amino acids (Gibco, 11140; Grand Island, NY, USA), 2 mM l-glutamine (Gibco, 25030; Grand Island, NY, USA), and 1 mM sodium pyruvate (Gibco, 11360; Grand Island, NY, USA) at 37°C in a 5% CO_2_ incubator.

### Purification of native Igl protein by affinity chromatography.

Native Igl protein from E. histolytica was purified as previously described (10). In general, the anti-Igl monoclonal antibody (MAb) EH3015 was bound to the CNBr-activated Sepharose 4B gel. Trophozoites (10^8^) of E. histolytica HM-1:IMSS in logarithmic growth phase were harvested and suspended in 50 mM Tris-HCl buffer (pH 8.3) containing 150 mM NaCl, 0.5% Nonidet P-40, and 5 mM EDTA disodium salt prior to sonication. Then, the soluble fraction was applied to the MAb EH3015-bound affinity column. After extensive washing, the native Igl protein was eluted with 0.2 N acetic acid (pH 3.0) and dialyzed in 20 mM Tris-HCl buffer (pH 8.0) immediately. The purity of the protein samples was confirmed by sodium dodecyl sulfate-polyacrylamide gel electrophoresis (SDS-PAGE) (see Fig. S3 in the supplemental material).

10.1128/msystems.01353-21.3 FIG S3 SDS-PAGE analysis of purified native Igl protein. A total of 3 μg of proteins was electrophoresed in a 10% gel under reducing conditions. Protein bands were visualized with Coomassie brilliant blue. M, Biostep prestained protein marker (Tanon, Shanghai, China); nIgl, purified native Igl protein. Download FIG S3, TIF file, 2.9 MB. Copyright © 2022 Zhao et al. 2022 Zhao et al. https://creativecommons.org/licenses/by/4.0/ This content is distributed under the terms of the Creative Commons Attribution 4.0 International license.

### Expression and purification of recombinant Igl protein.

Recombinant Igl protein was expressed and purified as previously described (13). Briefly, full-length Igl (GenBank accession no. AF337950.1) without glycosylphosphatidylinositol (GPI)-anchored sequences was cloned into pET-19b vectors (Novagen, Darmstadt, Germany). E. coli BL21 Star(DE3)pLysS competent cells (Weidibio, Shanghai, China) were transformed with the cloned plasmids. Bacteria were cultured in Luria-Bertani medium containing 100 μg/mL ampicillin. Isopropyl-β-d-thiogalactopyranoside (Amresco, Solon, OH, USA) was used for inducing recombinant Igl expression at a final concentration of 1 mM. The bacteria were harvested after incubation at 37°C for 3 h. The inclusion body was purified and refolded using the protein refolding kit (Novagen, Darmstadt, Germany). The refolded protein was further purified using the Ni-NTA (nitrilotriacetic acid) His-Bind resin kit (Novagen, Darmstadt, Germany). The purity of the protein samples was confirmed using SDS-PAGE (see Fig. S4).

10.1128/msystems.01353-21.4 FIG S4 SDS-PAGE analysis of purified recombinant Igl protein under reducing conditions. Protein bands were visualized with Coomassie brilliant blue. M, Biostep prestained protein marker (Tanon, Shanghai, China); lane 1, inclusion body before Ni-NTA chromatography; lanes 2 to 5, elution fractions of Ni-NTA chromatography containing purified recombinant Igl protein. Download FIG S4, TIF file, 0.9 MB. Copyright © 2022 Zhao et al. 2022 Zhao et al. https://creativecommons.org/licenses/by/4.0/ This content is distributed under the terms of the Creative Commons Attribution 4.0 International license.

### Pulldown assay and protein identification by mass spectrum.

The pulldown assay was performed following the instructions of the Pierce Pull-Down PolyHis Protein:Protein Interaction kit (Thermo Fisher Scientific, Rockford, IL, USA). In general, purified recombinant Igl protein (fusion protein with His_6_) was incubated with HisPur cobalt resin for 1 h on a rotating platform. The negative control was incubated with HisPur cobalt resin without Igl protein binding. Caco2 cells were harvested and solubilized using Pierce lysis buffer. Cell lysates were centrifuged at 12,000 × *g* for 5 min to obtain the clarified supernatants. Immobilized bait protein (Igl) or empty resin was incubated with prey protein (cell lysate) at 4°C. The spin column was then washed five times before the elution of the immune complex. Proteomics analysis of the eluted fractions was performed. Proteins identified in the negative control were considered nonspecific or background interactors. For determined interactors, KEGG pathway enrichment analysis and Cytoscape network analysis were conducted to investigate the links between the significantly enriched top 20 pathways and the top 10 hub genes interacting with Igl.

### Sample preparation for DIA quantitative proteomics analysis.

CHO and Caco2 cells were seeded in 6-well culture plates (5 × 10^5^ cells/well). After adhesion, cells were treated with purified native Igl protein (10 μg/mL) for 24 h or trophozoites of E. histolytica HM-1:IMSS (2.4 × 10^5^ cells/well) for 60 min. Before collection, cells were stained by trypan blue to ensure sufficient cell survival rate (>99%). After washing with 1× phosphate-buffered saline (PBS) three times to remove trophozoites, cells were collected and lysed in 0.1× PBS containing a protease inhibitor cocktail (Sigma-Aldrich, P8340; St. Louis, MO, USA). The concentrations of soluble proteins were measured using the Pierce bicinchoninic acid (BCA) protein assay kit (Thermo Fisher Scientific, Rockford, IL, USA). The samples were reacted with 10 mM dithiothreitol at 37°C for 1 h and then placed at room temperature for 30 min with the addition of 20 mM iodoacetamide. Later, four times the volume of cold acetone was added to the samples, and the samples were stored at −80°C overnight. After centrifugation at 12,000 × *g* for 15 min, the precipitates were dissolved in 50 mM ammonium bicarbonate solution. After protein digestion with trypsin (1:20 [wt/wt]; Promega, Beijing, China) overnight, the peptides were desalted using a Sep-Pak column (Waters, Framingham, MA, USA) according to the manufacturer’s instructions.

### Chromatography and mass spectrometry.

Peptides (200 ng) were analyzed using NanoElute (Bruker Daltonics, Billerica, MA, USA) liquid chromatography for LC-MS/MS analysis, using 25-cm by 75-μm, C_18_, 1.6-μm chromatographic columns (AUEOEA series) for LC separation. Phase A was 0.1% formic acid, and phase B was 0.1% formic acid in acetonitrile. The flow rate was set to 300 nL/min, and the analysis time was 1 h. The gradient was set as follows: phase B increased from 2% to 22% within 0 to 45 min, increased to 37% within 5 min, and increased to 37% within 5 min, 80%, with flushing for 5 min. All files were analyzed by a TIMS-TOF Pro mass spectrometer (Bruker Daltonics, Billerica, MA, USA) with a nano-electrospray ion source. The conditions of mass spectrometry were set as follows: the scanning range was 100 to 1,700 *m/z*, and the wavy range was set to 0.7 to 1.3 V·s/cm^2^. The single-cycle acquisition time was 1.16 s, the intensity threshold was 5,000, and the accumulation and release time was set as 100 ms. The voltage of the ion source was 1,500 V. The auxiliary gas was set to 3 L/min, and the ion source temperature was set to 180°C. DIA analysis was performed for all the sample groups.

### Protein quantification and bioinformatic analysis.

The MS data were analyzed using PEAKS Online 1.5 for label-free protein quantification (Bioinformatics Solutions Inc., Waterloo, Canada). The data library was downloaded from the Swiss-Prot database of human proteins (20 August 2020; 20,375 entries). The following parameters were used: trypsin digestion; fixed modification C; precursor mass error tolerance, 15 ppm; fragment mass error tolerance, 0.05 Da; collision cross section error tolerance, 0.05. The top 3 peptide areas were used for the protein quantification. Proteins with overall missing values greater than 50% were deleted, and the remaining empty values were filled with a random number between 0 and the value of the smallest area. The *t* test and 1.5-fold up-and-down volcano maps were used to screen for differentially expressed proteins. The significance level was 0.05 with FDR correction, and the statistical analysis was performed using the R package (version 4.1.1). GO and KEGG enrichment analyses were conducted using the R package. The enriched terms and pathways with *P* values of <0.05 were considered significant.

### Western blotting.

Western blotting was performed to confirm the results of quantitative proteomics. Briefly, protein samples of CHO cells and Caco2 cells stimulated with E. histolytica or Igl were separated on 10% polyacrylamide gels and then electrotransferred to polyvinylidene difluoride membranes (General Electric Co., Schenectady, NY, USA). After blocking with bovine serum albumin (5% in PBS), membranes were incubated with the following primary antibodies: anti-β-actin antibody (Abcam, Ab8227; Cambridge, United Kingdom), anti-PKM2 monoclonal antibody (Cell Signaling Technology, catalog no. 4053; Boston, MA, USA), and anti-LC3A/B antibody (Cell Signaling Technology, catalog no. 12741; Boston, MA, USA). Horseradish peroxidase (HRP)-conjugated goat anti-rabbit IgG(H+L) (Abcam, Ab6721; Cambridge, United Kingdom) was used as the secondary antibody. Proteins were detected using an enhanced chemiluminescence (ECL) Western blotting substrate kit (Tanon, Shanghai, China).

### Animal model for amoebic colitis.

Eight-week-old male C3H/HeNCrl mice were obtained from Beijing Vital River Laboratory Animal Technology Company. Amoebic colitis was induced by directly inoculating 1 × 10^6^ axenic E. histolytica SAW755CR trophozoites into the cecum (*n *= 6), and the control group received the same volume of medium without trophozoites (*n *= 4). Six days after inoculation, the cecum tissues were harvested and fixed in 4% paraformaldehyde, followed by paraffin embedding. Tissue sections were stained with hematoxylin and eosin or periodic acid-Schiff (PAS) stain for histopathological analysis. Furthermore, immunohistochemical staining was performed using anti-PKM2 monoclonal antibody (Cell Signaling Technology, D78A4; Boston, MA, USA) to detect the expression level of PKM2 in the cecum.

All animal experiments were performed in strict accordance with the guidelines of the Regulations for the Administration of Affairs Concerning Experimental Animals (1988.11.1) and approved by the Institutional Animal Care and Use Committee (permit no. 20160225-097). All efforts were made to minimize animal suffering.

### Fluorescence imaging of host cells incubated with trophozoites.

CHO cells were coincubated with trophozoites (at a ratio of 2:1) at 37°C for 30 min. The cells were then fixed in 4% paraformaldehyde solution and permeabilized in 0.1% Triton X-100 for 5 min. Rabbit anti-PKM2 monoclonal antibody (Cell Signaling Technology, D78A4; Boston, MA, USA) and mouse MAb EH3015 against Igl were used as primary antibodies. Alexa Fluor 488 goat anti-rabbit IgG(H+L) and 594 goat anti-mouse IgG(H+L) (Thermo Fisher Scientific, Rockford, IL, USA) were used as secondary antibodies. After counterstaining with 0.5 μg/mL 4′,6-diamidino-2-phenylindole (DAPI), cells were observed under a laser confocal microscope (Zeiss, Jena, Germany).

### Data availability.

The raw proteomics data are available at the jPOST database under accession number JPST001507 (PXD031959).

## Supplementary Material

Reviewer comments
