# Supplementary figures and images for: Quantitative Proteomics Reveals Metabolic Reprogramming in Host Cells Induced by Trophozoites and Intermediate Subunit of Gal/GalNAc Lectins from Entamoeba histolytica
Source: mSystems. 2022 Mar 28;7(2):e01353-21. doi: 10.1128/msystems.01353-21 (PMC9040881; doi:10.1128/msystems.01353-21)

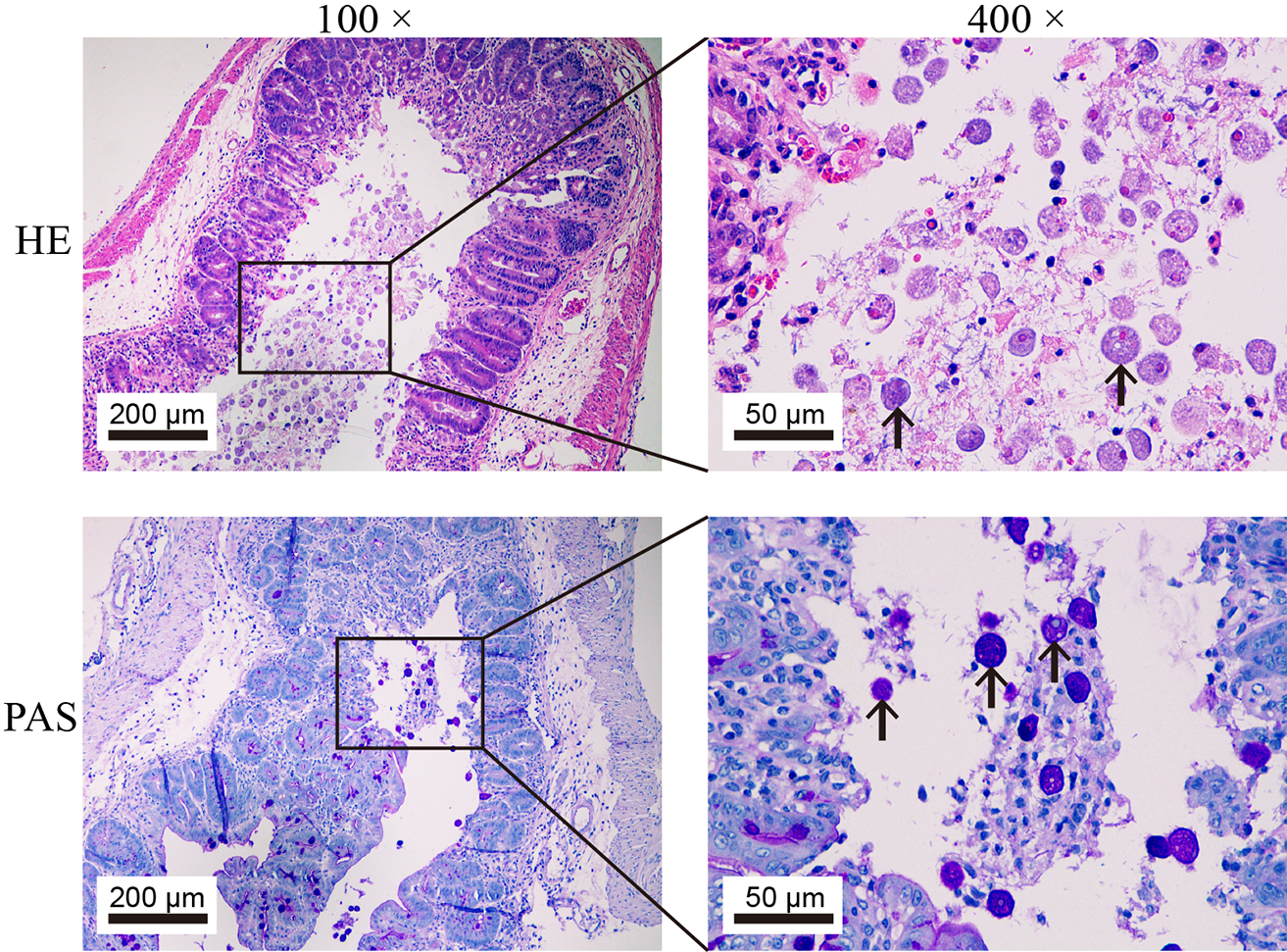

Supplement: FIG S1 [file msystems.01353-21-s0001.tif]

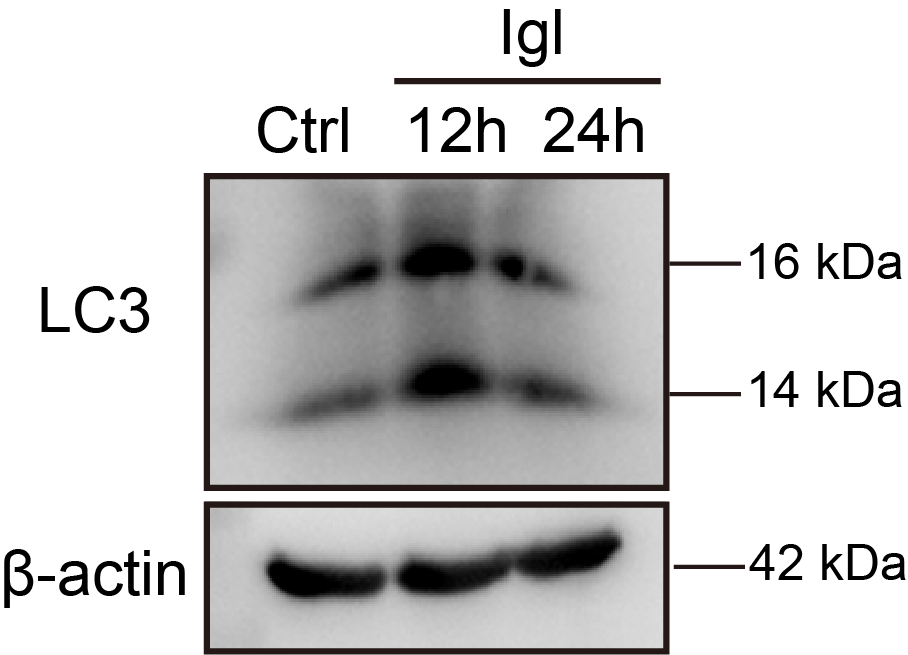

Supplement: FIG S2 [file msystems.01353-21-s0002.tif]

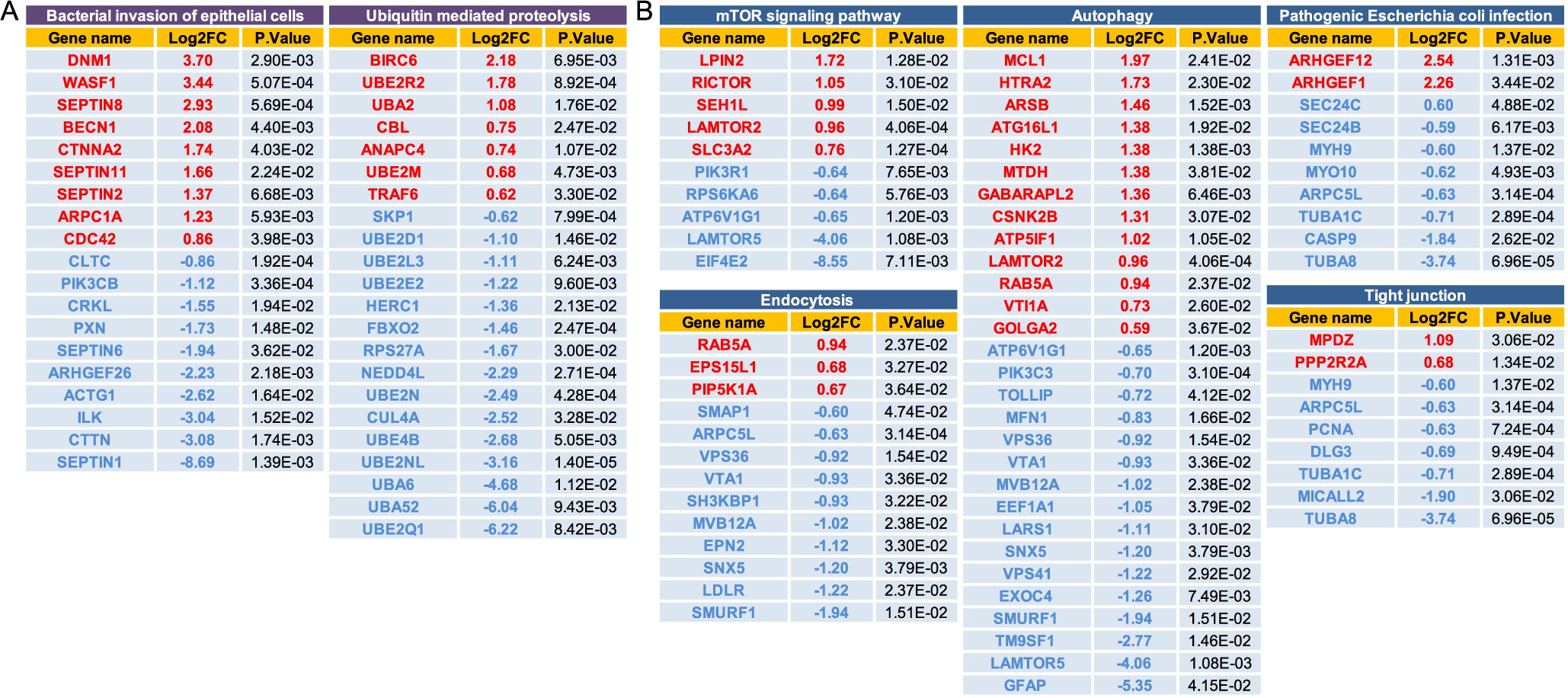

Supplement: FIG S5 [file msystems.01353-21-s0005.tif]

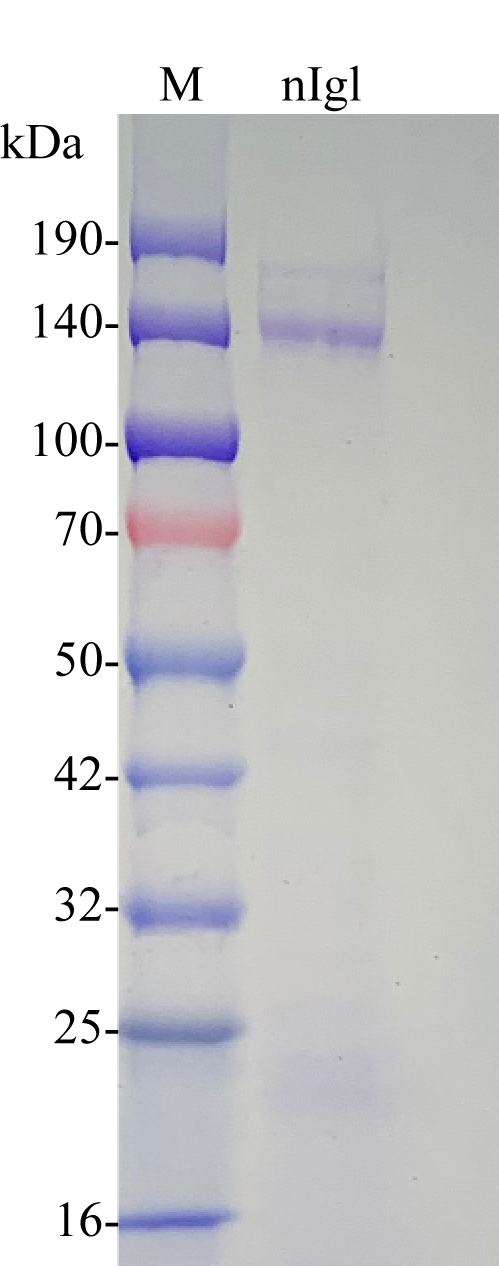

Supplement: FIG S3 [file msystems.01353-21-s0003.tif]

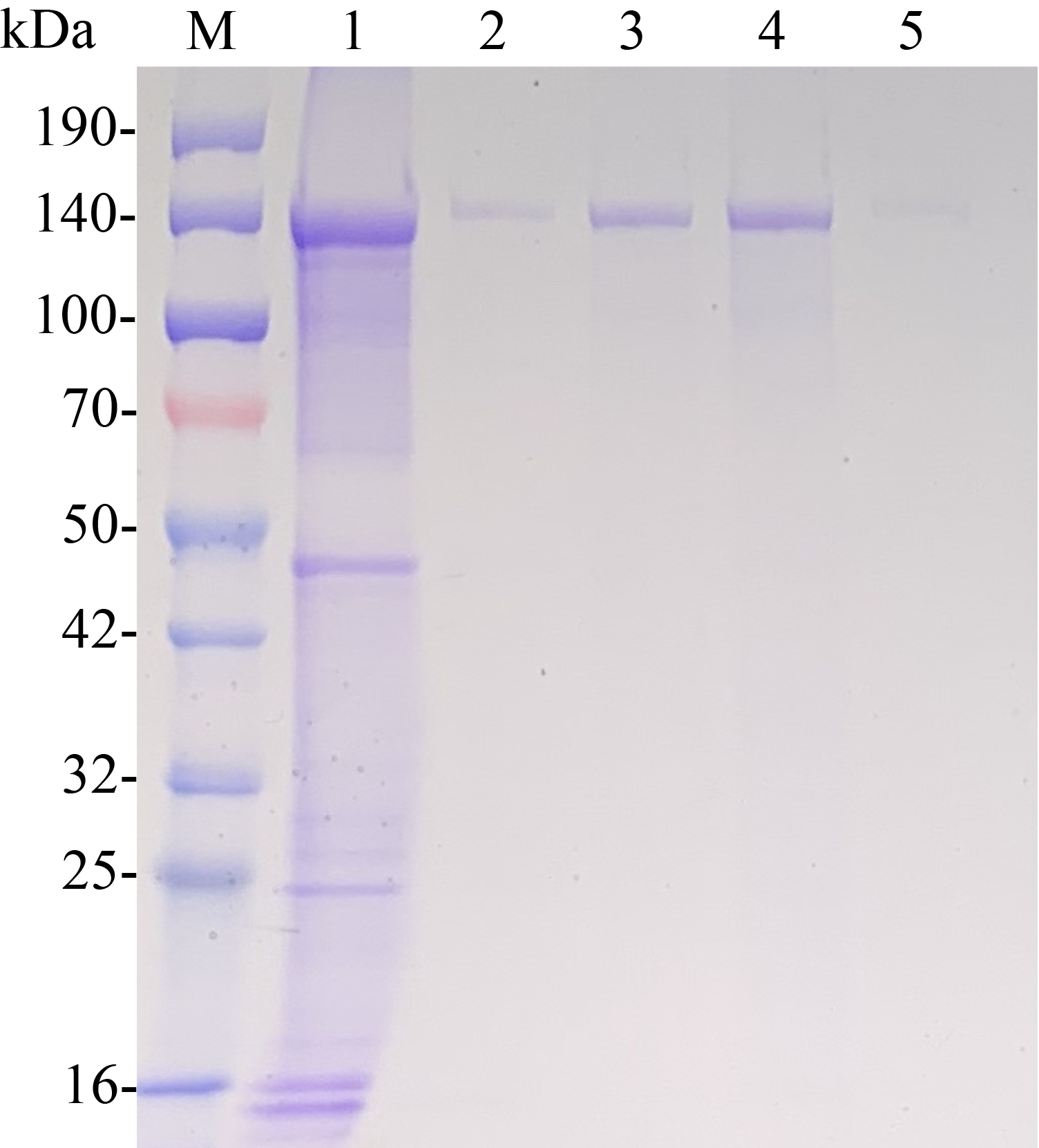

Supplement: FIG S4 [file msystems.01353-21-s0004.tif]
